# The associations between social support and mental health among Chinese immigrant pregnant and parenting women

**DOI:** 10.1186/s12884-024-06765-9

**Published:** 2024-09-06

**Authors:** Grace Tian, Natalia M. Rojas, Jennifer M. Norton, R. Gabriela Barajas-Gonzalez, Jacqueline Montesdeoca, Bonnie D. Kerker

**Affiliations:** 1 Center for Early Childhood Health and Development, Population Health, NYU Grossman School of Medicine, New York, NY USA; 2 Department of Population Health, NYU Grossman School of Medicine, New York, NY USA

**Keywords:** Social support, Chinese immigrant women, Anxiety, Depression, Pregnancy status

## Abstract

**Background:**

While it is recognized that social support can alleviate mental health symptoms, this relationship is not well-understood among Chinese pregnant and parenting immigrants in the United States. This study aims to bridge this gap by exploring the relationships between different types of social support and women’s anxiety and depression, and examining how these associations vary with pregnancy status.

**Methods:**

Data were obtained from a cross-sectional survey conducted in Simplified Chinese or Mandarin between March-June 2021 among 526 women who were pregnant and/or parenting a child under five years. The Patient-Reported Outcomes Measurement Information System (PROMIS) Anxiety, Depression, and Social Support scales were used to measure anxiety, depression, and social support levels. Descriptive statistics, *t*-tests, chi-square tests, and Pearson’s correlations were employed for analysis. Hierarchical regression was conducted to investigate the main and interaction effects of social support types and pregnancy status on mental health outcomes.

**Results:**

Compared to non-pregnant women, pregnant women reported higher mean scores for anxiety (non-pregnant: 55, pregnant: 59, *p* < 0.01) and depression (non-pregnant: 54, pregnant: 56, *p* = 0.02). Instrumental support displayed a significant main effect in relation to anxiety (β=-0.13, *p* = 0.01) and depression (β=-0.16, *p* < 0.01); emotional support exhibited a significant main effect solely on depression (β=-0.13, *p* = 0.01). Notably, the interaction effects between pregnancy status and both instrumental (β=-0.28, *p* = 0.01) and emotional support (β=-0.42, *p* < 0.01) were significant for anxiety. In contrast, informational support did not exhibit a significant impact on either anxiety or depression.

**Conclusions:**

The findings indicate that tailoring support to the cultural context is crucial, especially for pregnant women in this Chinese immigrant community, with instrumental and emotional support being particularly beneficial in mitigating maternal anxiety.

**Supplementary Information:**

The online version contains supplementary material available at 10.1186/s12884-024-06765-9.

## Background

Pregnant and parenting women represent a vulnerable group at elevated risk of experiencing adverse mental health outcomes [1–3]. Extensive research consistently highlights the prevalence of mental health concerns during the pregnancy and postpartum periods [4–6]. These critical phases are characterized by significant physical, emotional, and social changes, contributing to heightened mental health concerns [7–10]. Additionally, postpartum depressive symptoms can impact children’s neurodevelopment and overall health [11–14], and such symptoms and effects can last beyond the initial year after childbirth [15–17].

Chinese immigrants constitute one of the largest immigrant groups in the United States (U.S.) [18]. Underserved Chinese immigrant women in the U.S. encounter multiple challenges, including language barriers, economic instability, and social isolation [19–21], a significant risk factor for depression [22, 23]. The coronavirus disease 2019 (COVID-19) pandemic has further heightened mental health concerns for these women, due to stress and anxiety over health and economic uncertainties [24]. Research has demonstrated that Chinese immigrant mothers with young children exhibit higher levels of depressive symptoms compared to non-immigrant mothers [25]. However, limited research has specifically examined the mental health challenges of Chinese immigrant pregnant women in the United States.

The significant impact of culturally-relevant social support on mental health outcomes among immigrant populations has been consistently underscored in research [26–29]. Social support is a multidimensional construct encompassing instrumental support, involving tangible aid; informational support, providing guidance and knowledge; and emotional support, offering empathy and comfort during times of stress [30, 31]. Studies across different countries highlight distinct impacts of different types of social support on mental health outcomes [32–34], particularly given women’s distinctive needs for various types of support during pregnancy and different motherhood stages [35–38]. For Chinese immigrant women, social support has been associated with lower mental health symptoms [39–41]. However, the associations between different types of social support and mental health outcomes among Chinese immigrant pregnant women and mothers remains unclear.

### The current study

This study will address these gaps and pursue the following aims among a sample of Chinese pregnant women and mothers of young children: (Aim 1) to examine potential differences in levels of anxiety and depression among pregnant women and mothers, (Aim 2) to explore the associations between different types of social support and anxiety and depression, and (Aim 3) to investigate whether these associations vary by pregnancy status. By examining these factors, this study has the potential to inform the development of tailored interventions that address anxiety and depression among underserved Chinese immigrant women.

## Method

### Study design

This study is part of the “Together Growing Strong” initiative in Sunset Park, Brooklyn, which supports families with young children. While Sunset Park, a vibrant immigrant community, has tight-knit families, it grapples with issues like worker exploitation, low wages, and overcrowded housing.

Using a mixed-methods approach, we conducted both quantitative surveys and qualitative interviews. Materials were available in English, Spanish, and Chinese (Simplified Chinese and Mandarin) to cater to community preferences. We used a convenience sample to recruit participants from health centers, social service locations, and texting platforms. Furthermore, participants could choose to self-administer the survey online or complete the survey over the phone with a trained interviewer. Ethical approval for this research has been granted by the Institutional Review Board of NYU Grossman School of Medicine (reference number: s20-01944).

### Participants

Study participants were women aged 18 years or older and either pregnant or parenting a child under 5 years old. As an incentive, participants received a gift card worth $25 upon survey completion. The current analysis is based on the quantitative data from a subset of participants who completed the survey in Simplified Chinese or Mandarin between March and June 2021. To ensure linguistic and cultural consistency, only participants who completed the survey in Simplified Chinese or Mandarin were included in this analysis (*n* = 526). Of the 145 women excluded from the analysis, fifteen took the survey in English and identified as Chinese ethnicity, of whom 8 were born outside the U.S.

### Measures

#### Dependent variables: PROMIS anxiety and depression

Participants completed the Patient-Reported Outcomes Measurement Information System^®^ (PROMIS^®^) Short-Form (SF) v1.0 – Anxiety and the PROMIS SF v1.0 – Depression [42], assessing anxiety and depression symptoms. Both scales used a 5-point Likert scale to rate symptoms over seven days. The Anxiety scale comprised four items, including aspects, such as “I felt fearful” and “I felt uneasy.” The Depression scale also had four items capturing self-reported feelings, such as “I felt worthless” and “I felt hopeless.” Total scores for anxiety and depression were converted to standardized T-scores, with a mean of 50 and a standard deviation of 10, based on a calibration sample from the U.S. general population. Higher T-scores indicated greater levels of anxiety or depression [43–45]. Severity levels for anxiety and depression were categorized based on T-scores, with 55-59.9 representing mild symptoms, 60-69.9 representing moderate symptoms, and ≥ 70 representing severe symptoms [46]. Previous research has demonstrated sufficient linguistic equivalence and cross-cultural validity of the Chinese version of the PROMIS SF [47–49].

#### Independent variable

##### PROMIS social support

To assess social support, the PROMIS Short Form v2.0 Informational, Instrumental, and Emotional Support scales were utilized [42]. These scales measure different dimensions of social support, with statements such as “I have someone to give me good advice about a crisis if I need it.” (informational support), “Do you have someone to help you if you are confined to bed?” (instrumental support), and “I have someone who will listen to me when I need to talk.” (emotional support). Participants responded to each item using a five-point Likert scale, with response options ranging from 1 (never) to 5 (always). Total scores for each dimension of social support were converted to standardized T-scores, with a mean of 50 and a standard deviation of 10, based on a calibration sample from the U.S. general population. Higher T-scores indicated greater levels of support [43, 45]. The PROMIS Social Support scales have been translated and psychometrically validated in Chinese [49, 50].

#### Pregnancy status

Pregnancy status was based on the response to the question “Are you currently pregnant?” Women were categorized as “pregnant” if they were currently pregnant regardless of whether they also had other children. Women were categorized as “not pregnant” if they were not currently pregnant and had at least one child aged less than five years.

#### Control variables

Covariate selection involved two steps. First, we identified potential covariates through a literature review of factors relevant to mental health and social support among immigrant populations. These included age, marital status, financial difficulties, general health status, education level, language spoken, and years living in the U.S. [51–56]. Second, to ensure model parsimony while accounting for relevant factors, we conducted bivariate analyses to determine significant associations with our outcomes and independent variables (Supporting Table 1). Covariates showing significant associations (*p* ≤ 0.05) in bivariate analyses were included in the multivariable models. These were: age (18–29 vs. 30 or above), marital status (single, divorced, widowed vs. married or living with a partner), having difficulties paying bills (no difficulty at all vs. any level of difficulty), and general health (poor or fair vs. good, very good or excellent). Education level, language spoken at home, and years living in the U.S. did not show significant associations with our primary outcomes or independent variables and were therefore not included in the final models.

### Statistical analysis

First, descriptive statistics were used to calculate the frequencies and mean scores for all analyzed variables. *t*-tests and Chi-Square tests were also used to examine differences between pregnant and non-pregnant women regarding the study variables, anxiety and depression (Aim 1).

Second, *t*-tests were conducted to examine differences in anxiety and depression scores across levels of socio-demographic and health-related variables. Pearson’s correlations were run to investigate associations among three types of social support, anxiety, and depression to understand the underlying associations among continuous variables (i.e. informational support, instrumental support, emotional support, anxiety, and depression) and to test for collinearity.

Third, hierarchical regression was performed to examine the main and interaction effects of each type of social support and pregnancy status in relation to anxiety and depression scores. For each outcome, three models were constructed to explore the associations between different types of social support and mental health outcomes (Aim 2), including Model 1 (instrumental support), Model 2 (emotional support), and Model 3 (informational support). In each model, Step 1 included the control variables, pregnancy status, and the corresponding type of support. In Step 2, the interaction term between pregnancy status and the corresponding support was added to the model to examine whether the associations vary by pregnancy status (Aim 3). To reduce multicollinearity between the main effects and interactions, we mean-centered the continuous independent variables (i.e. informational support, instrumental support, and emotional support). The variance inflation factor values and condition index were examined and no multicollinearity was found.

To address multiple comparisons, we applied the Benjamini-Hochberg procedure to control the false discovery rate (FDR) for each outcome separately across the three models. Both original and FDR-adjusted p-values are reported, with statistical significance determined based on adjusted p-values (*p* ≤ 0.05). Finally, to interpret the significant two-way interactions in the regression models, simple slope analyses were conducted. Missing data within the utilized variables ranged from 3 to 10%, and all such cases with missing data were excluded from the analysis.

## Results

### General descriptive statistics and pregnancy differences

Table 1 shows the socio-demographic characteristics for the total sample (*n* = 526) and by pregnancy status (*n* = 100 pregnant; *n* = 426 non-pregnant). Among the total sample, the majority were aged 30 and above (72%) and married or living with a partner (90%), and experienced difficulties paying bills (73%). Over half had completed at least a high school degree (57%). Of those not born in the U.S. (*n* = 423), 58% had lived in the U.S. for less than 10 years. Only 7% of the total sample reported speaking English at home. Although the pregnant and non-pregnant groups were similar in terms of education, marital status, economic difficulties, and immigration-related factors, they differed significantly in age. A lower percentage of pregnant women (59%) were aged 30 and above than the non-pregnant group (75%) (*p* < 0.01).

**Table 1:** Descriptive characteristics for the full sample and by pregnancy status

Variables	*n* ^a^	Total	Not Pregnant	Pregnant	Test Statistic		
		*n* (%)	*n* (%)	*n* (%)	*p*-value^b^	df	χ²
**Age**: 30 and above	483	346 (72%)	289 (75%)	57 (59%)	0.00*	1	9.12
**Education**: High school degree and above	488	279 (57%)	224 (57%)	55 (59%)	0.86	1	0.03
**Marital Status**: Married/Living with Partner	509	460 (90%)	377 (91%)	83 (86%)	0.21	1	1.57
**Have difficulties in paying bills or buying something**: Any level of difficulty	462	335 (73%)	265 (72%)	70 (76%)	0.47	1	0.53
**Years living in the U.S.**^c^: Less than 10 years	423	246 (58%)	189 (56%)	57 (65%)	0.20	1	1.67
**Speaks English at home**: Yes	526	38 (7%)	29 (7%)	9 (9%)	0.58	1	0.30
**General health**: Good and above	508	435 (86%)	355 (87%)	80 (80%)	0.10	1	2.66
		**Mean [CI]**	**Mean [CI]**	**Mean [CI]**	**p-value** ^b^	**df**	**t-value**
**PROMIS Informational Support**	507	43 [43; 44]	43 [42; 44]	44 [42; 45]	0.82	165	-0.23
**PROMIS Instrumental Support**	504	47 [46; 48]	47 [46; 48]	48 [47; 50]	0.10	150	-1.68
**PROMIS Emotional Support**	505	44 [43; 45]	44 [43; 45]	44 [43; 46]	0.87	178	-0.16
**PROMIS Anxiety**	516	56 [55; 57]	55 [54; 56]	59 [57; 61]	0.00*	152	-3.63
**PROMIS Depression**	517	54 [53; 55]	54 [53; 55]	56 [54; 58]	0.02*	159	-2.27
**Number of children**	522	1.64 [1.56; 1.73]	1.78 [1.69; 1.88]	1.02 [0.86; 1.19]	0.00*	162	7.92

^a^Of *N* = 526

^b^*p*-value comparing not pregnant and pregnant groups. Differences in continuous variables were detected by *t*-tests for independent samples. Differences in categorical variables were detected by χ2 tests

^c^Among those not born in the U.S

*Significant at the *p* ≤ 0.05 level

The majority of the sample (86%) reported their health as good, very good or excellent. The mean scores for informational, instrumental, and emotional support were 43, 47, and 44, respectively. Overall, the mean anxiety score was 56, with 37% of women having a score at or above 60, indicating moderate to severe anxiety. Overall, the mean depression score was 54, with 31% of women having a score at or above 60, indicating moderate to severe depression. No significant differences were observed between pregnant and non-pregnant groups across general health and mean levels of support. However, compared to non-pregnant women, pregnant women reported higher mean scores for anxiety (non-pregnant: 55, pregnant: 59, *p* < 0.01) and depression (non-pregnant: 54, pregnant: 56, *p* = 0.02).

### Correlations between support, anxiety, and depression

Moderate to strong positive correlations were found between informational and emotional support (*r* = 0.78, *p* < 0.01), informational and instrumental support (*r* = 0.53, *p* < 0.01), and instrumental and emotional support (*r* = 0.58, *p* < 0.01). In addition, instrumental support and emotional support were negatively correlated with anxiety (instrumental: *r*=-0.19, *p* < 0.01; emotional: *r*=-0.24, *p* < 0.01) and depression (instrumental: *r*=-0.13, *p* < 0.001; emotional: *r*=-0.18, *p* < 0.01). No significant relationship was found between informational support and anxiety (*r*=-0.05, *p* = 0.28) or depression (*r*=-0.08, *p* = 0.08).

### Effect of social support and pregnancy status on anxiety and depression

#### Anxiety

Table 2 presents the regression analysis results for the associations between pregnancy status and social support, and anxiety. To account for multiple comparisons, both unadjusted and Benjamini-Hochberg adjusted p-values are reported. Statistical significance was determined based on the adjusted p-values, with *p* ≤ 0.05 considered significant. The majority of our findings remained statistically significant after this adjustment, enhancing the reliability of our results while maintaining sensitivity to potentially important relationships in this exploratory study. As seen in Model 1 (*N* = 406), Step 1 indicated that the main effects of instrumental support and pregnancy status were significantly related to anxiety (instrumental support: β=-0.13, *p* = 0.01, adjusted *p* = 0.02; pregnancy status: β = 2.19, *p* = 0.02, adjusted *p* = 0.03). Step 2 revealed a significant interaction effect between instrumental support and pregnancy status regarding anxiety (instrumental support X pregnancy: β=-0.28, *p* = 0.01, adjusted *p* = 0.02). The analysis conducted in Model 2 (*N* = 405), Step 1 found a statistically significant effect of pregnancy status (β = 2.15, *p* = 0.03, adjusted *p* = 0.05), but emotional support was not statistically related to anxiety (β=-0.07, *p* = 0.18, adjusted *p* = 0.22). Nevertheless, adding the interaction terms between emotional support and pregnancy status in Step 2 revealed a significant interaction effect on anxiety (emotional support X pregnancy: β=-0.42, *p* < 0.01, adjusted *p* < 0.01). In Model 3 (*N* = 403), focusing on informational support as the main independent variable, Step 1 results mirrored the findings of the bivariate analysis, indicating no significant association between informational support and anxiety (β = 0.02, *p* = 0.67, adjusted *p* = 0.67); in Step 2, no significant interaction effect was observed for anxiety (informational support X pregnancy: β=-0.14, *p* = 0.30, adjusted *p* = 0.42) (see Supporting Table 2).

**Table 2:** Multivariable analysis: examining *anxiety* as an outcome

Independent and Control Variables	Anxiety - Model 1 (*N* = 406)										Anxiety - Model 2 (*N* = 405)									
	(Independent Variable: Instrumental Support)										(Independent Variable: Emotional Support)									
	Step 1 (Main Effect)					Step 2 (Interaction Effect)					Step 1 (Main Effect)					Step 2 (Interaction Effect)				
	β	*p*-value	Adj. *p*-value	L95% CI	U95% CI	β	*p*-value	Adj. *p*-value	L95% CI	U95% CI	β	*p*-value	Adj. *p*-value	L95% CI	U95% CI	β	*p*-value	Adj. *p*-value	L95% CI	U95% CI
**Age**: 30 or older ^a^	1.46	0.11	0.13	-0.31	3.22	1.36	0.13	0.16	-0.39	3.11	1.82	**0.04ˆ**	0.06	0.06	3.59	1.9	**0.03ˆ**	0.05	0.15	3.65
**Marital Status**: Separated/Divorced/Widowed/Single ^b^	3.00	**0.02ˆ**	0.03*	0.42	5.58	3.37	**0.01ˆ**	0.02*	0.79	5.95	3.48	**0.01ˆ**	0.02*	0.89	6.07	3.66	**0.01ˆ**	0.02*	1.09	6.23
**Have difficulties in paying bills or buying something**: Any level of difficulty ^c^	5.26	**0.00ˆ**	0.00*	3.41	7.10	5.19	**0.00ˆ**	0.00*	3.36	7.02	5.65	**0.00ˆ**	0.00*	3.84	7.46	5.56	**0.00ˆ**	0.00*	3.77	7.35
**General Health**: Poor and fair ^d^	7.57	**0.00ˆ**	0.00*	5.47	9.67	7.56	**0.00ˆ**	0.00*	5.48	9.64	7.75	**0.00ˆ**	0.00*	5.6	9.90	7.67	**0.00ˆ**	0.00*	5.54	9.80
**Pregnancy Status**: Pregnant ^e^	2.19	**0.02ˆ**	0.03*	0.32	4.06	2.48	**0.01ˆ**	0.02*	0.61	4.34	2.15	**0.03ˆ**	0.05*	0.26	4.04	2.28	**0.02ˆ**	0.04*	0.40	4.16
**Support** ^g^	-0.13	**0.01ˆ**	0.02*	-0.22	-0.04	-0.07	0.16	0.17	-0.17	0.03	-0.07	0.18	0.22	-0.18	0.03	-0.01	0.85	0.85	-0.12	0.10
**Interaction: Support**^h^**X Pregnancy Status**: Pregnant ^f^						-0.28	**0.01ˆ**	0.02*	-0.49	-0.08						-0.42	**0.00ˆ**	0.00*	-0.70	-0.14

^a^Reference group: Ages18-29

^b^Reference group: Married or living with partner

^c^Reference group: No difficulty at all

^d^Reference group: Good and above

^e, f^Reference group: Non-pregnant

^g, h^The variable Support in Model 1 refers to Instrumental Support and in Model 2 refers to Emotional Support

ˆ Significant at *p* ≤ 0.05 level based on original *p*-value

* Significant at *p* ≤ 0.05 level based on adjusted *p*-value (Benjamini-Hochberg procedure)

**Fig. 1:**
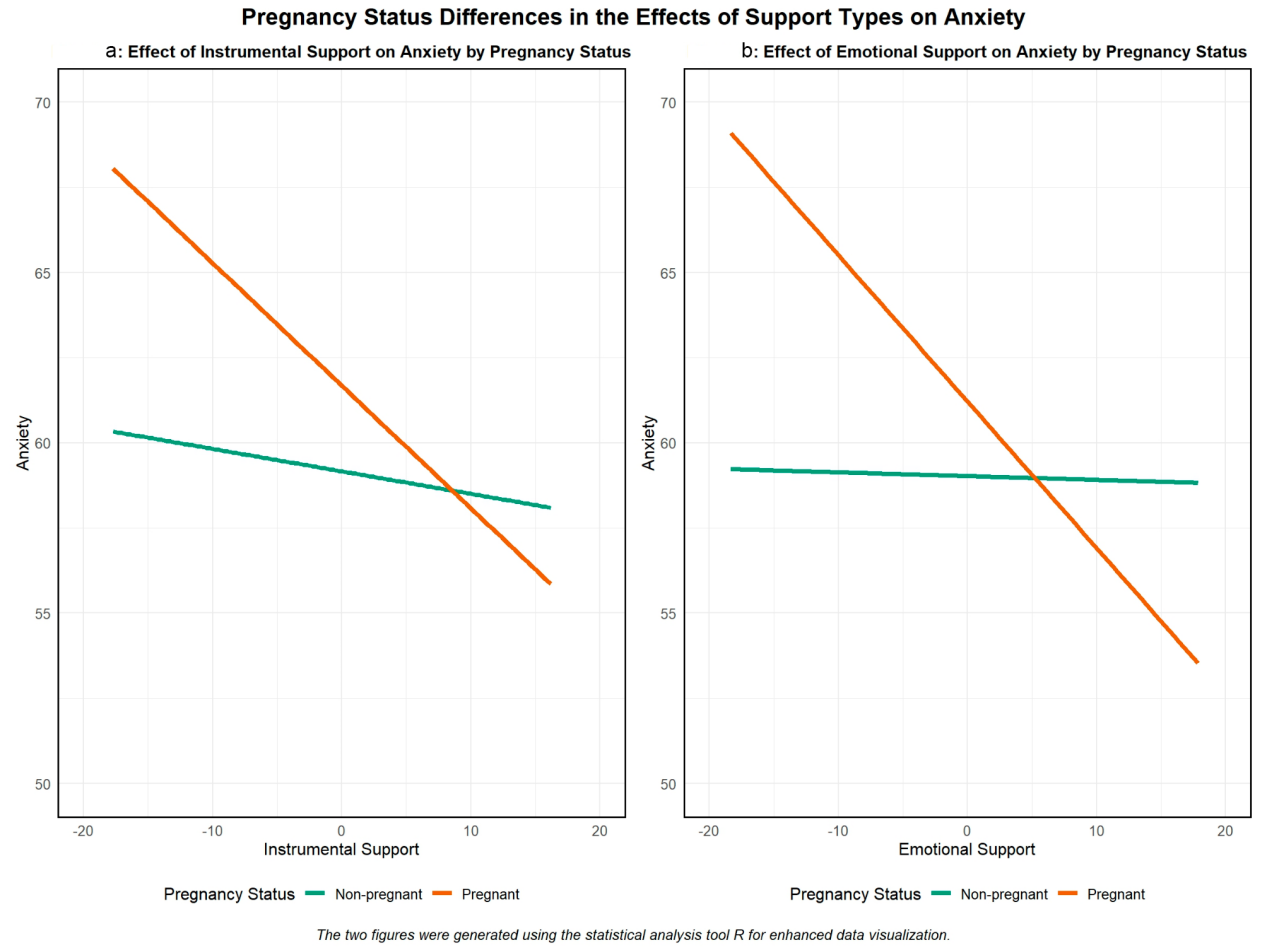
Pregnancy Status Differences in the Effects of Support Types **a** Instrumental and **b** Emotional on Anxiety

Post hoc simple slope analyses were conducted for the significant interactions. Figure 1a depicts that higher levels of instrumental support were associated with lower levels of anxiety in the non-pregnant group (β=-0.07), but this relationship was stronger in the pregnant group (β=-0.35) (marginal difference = 0.28, *p* = 0.01). Figure 1b shows an even starker difference in the association between emotional support and anxiety, between the pregnant and non-pregnant groups, with a marginal difference of 0.42 (*p* < 0.01).

#### Depression

Table 3 displays the regression analysis results exploring the impact of pregnancy status and social support on depression. In contrast to the anxiety models, the hierarchical regression results suggested the main effect of pregnancy status was not significant in any of the three models.

**Table 3:** Multivariable analysis: examining *Depression* as an outcome

Independent and Control Variables	Depression - Model 1 (*N* = 405)										Depression- Model 2 (*N* = 405)									
	(Independent Variable: Instrumental Support)										(Independent Variable: Emotional Support)									
	Step 1 (Main Effect)					Step 2 (Interaction Effect)					Step 1 (Main Effect)					Step 2 (Interaction Effect)				
	β	*p*-value	Adj. *p*-value	L95% CI	U95% CI	β	*p*-value	Adj. *p*-value	L95% CI	U95% CI	β	*p*-value	Adj. *p*-value	L95% CI	U95% CI	β	*p*-value	Adj. *p*-value	L95% CI	U95% CI
**Age**: 30 or older ^a^	1.03	0.24	0.29	-0.67	2.73	0.98	0.26	0.31	-0.72	2.68	1.37	0.12	0.18	-0.34	3.09	1.40	0.11	0.18	-0.32	3.11
**Marital Status**: Separated/Divorced/Widowed/Single ^b^	3.24	**0.01ˆ**	0.02*	0.74	5.73	3.41	**0.01ˆ**	0.02*	0.90	5.92	3.79	**0.00ˆ**	0.00*	1.28	6.30	3.84	**0.00ˆ**	0.00*	1.33	6.36
**Have difficulties in paying bills or buying something**: Any level of difficulty^c^	5.36	**0.00ˆ**	0.00*	3.57	7.15	5.33	**0.00ˆ**	0.00*	3.54	7.12	5.88	**0.00ˆ**	0.00*	4.12	7.63	5.85	**0.00ˆ**	0.00*	4.09	7.61
**General Health**: Poor and fair ^d^	7.16	**0.00ˆ**	0.00*	5.13	9.18	7.15	**0.00ˆ**	0.00*	5.13	9.18	7.30	**0.00ˆ**	0.00*	5.21	9.38	7.27	**0.00ˆ**	0.00*	5.18	9.35
**Pregnancy Status**: Pregnant ^e^	0.61	0.51	0.51	-1.2	2.41	0.74	0.42	0.46	-1.08	2.55	0.48	0.61	0.61	-1.36	2.32	0.52	0.58	0.70	-1.32	2.36
**Support** ^g^	-0.16	**0.00ˆ**	0.00*	-0.25	-0.08	-0.14	**0.01ˆ**	0.02*	-0.23	-0.04	-0.13	**0.01ˆ**	0.02*	-0.24	-0.03	-0.11	**0.04ˆ**	0.08	-0.22	0.00
**Interaction: Support**^h^**X Pregnancy Status**: Pregnant ^f^						-0.13	0.20	0.28	-0.33	0.07						-0.13	0.34	0.48	-0.41	0.14

^a^Reference group: Ages18-29

^b^Reference group: Married or living with partner

^c^Reference group: No difficulty at all

^d^Reference group: Good and above

^e, f^Reference group: Non-pregnant

^g, h^The variable Support in Model 1 refers to Instrumental Support and in Model 2 refers to Emotional Support

ˆ Significant at *p* ≤ 0.05 level based on original *p*-value

* Significant at *p* ≤ 0.05 level based on adjusted *p*-value (Benjamini-Hochberg procedure)

In Depression Models 1 (*N* = 405) and 2 (*N* = 405), instrumental support (β=-0.16, *p* < 0.01, adjusted *p* < 0.01) and emotional support (β=-0.13, *p* = 0.01, adjusted *p* = 0.02) were statistically significantly related to depression. No significant interaction effects were found between any type of support and pregnancy status when considering depression, suggesting that the effect of support on depression does not differ by pregnancy status. Consistent with the results from the bivariate analysis, no significant influence of informational support on depression was found (see Supporting Table 3).

## Discussion and conclusion

### Mental health challenges

This study reveals that low-income Chinese immigrant mothers endorsed a range of mental health challenges, as evidenced by the survey results indicating a substantial prevalence of moderate to severe anxiety and depression among Chinese immigrant pregnant women and mothers of young children. These findings align with prior research highlighting the heightened risk of anxiety and depression during pregnancy among Chinese women [57, 58].

Further, while regression analyses indicated that pregnancy status did not have a significant effect on depression scores, pregnancy status was a significant independent variable for anxiety models even when controlling for social support. This indicates the unique contribution of pregnancy to heightened anxiety levels among Chinese immigrant women. The COVID-19 pandemic may have exacerbated perinatal mental health challenges, particularly in immigrant communities [59]. It is crucial to identify and implement strategies, including enhancing social support, to alleviate maternal mental challenges, particularly addressing perinatal anxiety.

### The importance of support in mitigating mental health symptoms: cultural specificity, immigration factors, and pregnancy status

Our findings underscore the importance of support in mitigating mental health symptoms and highlight the nuanced roles of different types of support. Specifically, instrumental support exhibited a significant main effect on both anxiety and depression; emotional support demonstrated a significant main effect exclusively on depression, whereas informational support did not manifest any significant influence. Notably, the relationships between support types and anxiety/depression might be culturally specific to our study’s group of Chinese predominantly immigrant mothers. In East Asian cultural contexts, tangible/instrumental support providing practical resources has been shown to be prioritized [60]. Similar to our findings, Chen et al.‘s [39]. study in China suggested increased instrumental/emotional support was significantly associated with reduced postpartum depression over time, with informational support lacking significance. However, studies involving women from diverse cultural backgrounds, including Turkish [61], Australian [33], Jordanian [62], and American [38], populations, produced mixed results, underscoring the potential cultural specificity in the relationships between support types and women’s mental health conditions.

The immigration experience of our participants likely plays a crucial role in shaping the relationships between social support and mental health outcomes. Given that a substantial proportion of our sample reported not speaking English at home it is important to consider how these sociolinguistic factors might influence our findings. Recent immigrants often face challenges in accessing and utilizing social support due to limited social networks, unfamiliarity with local systems, and language barriers [63, 64]. These factors may explain the strong association we found between instrumental support and mental health outcomes, as tangible assistance becomes particularly crucial for those navigating a new environment and healthcare system, especially during pregnancy and early motherhood [65]. Language barriers can limit access to and comprehension of health information, potentially reducing the effectiveness of informational support [64, 66], which aligns with our finding of non-significant effects for this type of support. Furthermore, the immigration experience may amplify the importance of emotional support in combating feelings of isolation and maintaining mental health [67], consistent with our observed association between emotional support and depression outcomes. These findings underscore the need for culturally sensitive and language-appropriate support interventions for Chinese immigrant mothers. Future research should explicitly examine how acculturation levels, length of residency, and language proficiency moderate the relationships between different types of social support and mental health outcomes in this population.

In addition, the relationships between support and women’s mental health may vary depending on pregnancy status. Limited research examines the intricate dynamics among various forms of support, pregnancy status, and mental health. To address this gap, our study investigated the interaction effect of different support types and pregnancy on women’s mental well-being. Notably, our findings showed an even greater association between instrumental and emotional support and lower anxiety symptoms among pregnant women, compared to non-pregnant women.

Our study used general measures of anxiety and depression (PROMIS scales) to allow comparison between pregnant and non-pregnant participants, including those past the postpartum period. Pregnancy- and postpartum-specific measures might capture perinatal anxiety and depression more accurately; however, recent research has demonstrated strong correlations between general measures like PROMIS and pregnancy-specific measures, suggesting that general scales can capture mental health aspects relevant to pregnancy [68]. We explored the relationships between these general measures and the perinatal-specific Edinburgh Postnatal Depression Scale (EPDS) in our sample among pregnant participants. Consistent with prior findings, our preliminary analysis suggests there may be strong positive correlations between PROMIS Anxiety T-scores and EPDS scores as well as between PROMIS Depression T-scores and EPDS scores, particularly among the pregnant and postpartum women in our study. Nevertheless, we acknowledge that pregnancy-specific measures might capture additional nuances of perinatal mental health. Scales such as the EPDS could provide insights into pregnancy-specific concerns that may interact differently with various types of social support. For instance, informational support might be particularly beneficial in alleviating pregnancy-specific anxieties about childbirth or infant care, even if it shows less association with general anxiety symptoms in our current findings. Future research employing both general and pregnancy-specific measures could elucidate how different types of social support might differentially impact general versus pregnancy-specific mental health outcomes in this population.

### Tailoring support to the cultural context: considering the type and implementation of support

Our findings emphasize the need to customize the type and implementation of support to align with cultural context. Traditional Chinese pregnancy practices involve dietary and activity constraints, especially avoiding cold foods and water [69, 70], suggesting potential benefits of instrumental support, such as meal preparation. Additionally, cultural differences influence how women seek support from various providers based on their relationship; a focus on emotional support within existing social relationships may be accepted in Asian cultures [71, 72], while accepting informational assistance about personal issues may be less favored [73]. Our study demonstrated how specific types of social support can differentially buffer against anxiety and depression among immigrant Chinese women. However, limited research has explored the effective implementation of different support types within cultural context and immigration-related factors. To take into account cultural sensitivity and individual preferences, future studies should investigate the relationship between the various providers of social support and Chinese immigrant women to explore effective implementation strategies.

### Conclusions

Our study has several limitations. Firstly, the cross-sectional design limits our ability to examine the temporal dynamics of these associations and determine the directionality of the relationships. Secondly, data collection took place during the COVID-19 pandemic, which may have introduced additional barriers to social support for the participants. Future studies should consider investigating the long-term effects of the pandemic on the mental health of immigrant mothers and its interaction with social support dynamics. Thirdly, the use of self-reported measures in our study introduces the possibility of response bias, and the scales used may not fully capture all the types of support desired by the participants, such as spiritual support. Additionally, the use of general rather than pregnancy-specific mental health measures may have limited our ability to capture some unique aspects of perinatal mental health in our pregnant participants. Future studies on pregnant and postpartum populations could benefit from incorporating pregnancy-specific measures, as they might capture a more nuanced understanding of mental health symptoms in this population.

This study highlighted the mental health challenges faced by low-income Chinese immigrant pregnant women and mothers, with pregnant women experiencing significantly higher levels of anxiety and depression. Social support, particularly instrumental and emotional support, played a crucial role in mitigating women’s anxiety and depression symptoms. Pregnancy status can influence the relationships between support types and mental health outcomes. Tailored interventions considering the specific support needs of pregnant women and mothers of young children can help address mental health concerns in the Chinese immigrant community. Further research should examine the appropriate implementation of each type of support and identify strategies to provide specific support among Chinese immigrant women.

## Electronic supplementary material

Below is the link to the electronic supplementary material.

Supplementary Material 1

## Data Availability

The datasets utilized and analyzed in this study are accessible upon reasonable request from the corresponding author.

## Abbreviations

COVID-19: coronavirus disease 2019
PROMIS: Patient-Reported Outcomes Measurement Information System
SF: Short-Form

## References

[1] Brown SM, Doom JR, Lechuga-Peña S, Watamura SE, Koppels T. Stress and parenting during the global COVID-19 pandemic. Child Abuse Negl. 2020;110(2):104699. 10.1016/j.chiabu.2020.104699. 32859394 10.1016/j.chiabu.2020.104699 PMC7440155

[2] Cameron EE, Joyce KM, Delaquis CP, Reynolds K, Protudjer JLP, Roos LE. Maternal psychological distress & mental health service use during the COVID-19 pandemic. J Affect Disord. 2020;276(765–774):765–74. 10.1016/j.jad.2020.07.081. 32736186 10.1016/j.jad.2020.07.081 PMC7370903

[3] Spinelli M, Lionetti F, Pastore M, Fasolo M. Parents’ stress and children’s psychological problems in families facing the COVID-19 outbreak in Italy. Front Psychol. 2020;11(1713). 10.3389/fpsyg.2020.01713. 10.3389/fpsyg.2020.01713 PMC7350926 32719646

[4] Munk-Olsen T, Laursen TM, Pedersen CB, Mors O, Mortensen PB. New parents and mental disorders. JAMA. 2006;296(21):2582. 10.1001/jama.296.21.2582. 17148723 10.1001/jama.296.21.2582

[5] Vesga-López O, Blanco C, Keyes K, Olfson M, Grant BF, Hasin DS. Psychiatric disorders in pregnant and postpartum women in the United States. Arch Gen Psychiatry. 2008;65(7):805. 10.1001/archpsyc.65.7.805. 18606953 10.1001/archpsyc.65.7.805 PMC2669282

[6] Dennis C-L, Heaman M, Vigod S. Epidemiology of Postpartum depressive symptoms among Canadian women: Regional and National results from a cross-sectional survey. Can J Psychiatry. 2012;57(9):537–46. 10.1177/070674371205700904. 23073031 10.1177/070674371205700904

[7] Vanstone M, Kandasamy S, Giacomini M, DeJean D, McDonald SD. Pregnant women’s perceptions of gestational weight gain: a systematic review and meta-synthesis of qualitative research. Matern Child Nutr. 2016;13(4). 10.1111/mcn.12374. 10.1111/mcn.12374 PMC6866018 27873484

[8] DeGroot DW, Sitler CA, Lustik MB, Langan KL, Hauret KG, Gotschall MH, Gehrich AP. The effect of pregnancy and the duration of postpartum convalescence on the physical fitness of healthy women: a cohort study of active duty servicewomen receiving 6 weeks versus 12 weeks convalescence. PLoS ONE. 2021;16(7):e0255248. 10.1371/journal.pone.0255248. 34320030 10.1371/journal.pone.0255248 PMC8318247

[9] McKeough R, Blanchard C, Piccinini-Vallis H. Pregnant and postpartum women’s perceptions of barriers to and enablers of physical activity during pregnancy: a qualitative systematic review. J Midwifery Women’s Health. 2022;67(4):448–62. 10.1111/jmwh.13375. 35621324 10.1111/jmwh.13375

[10] Banker JE, Goldenson D. Clinical application of narrative therapy in the treatment of perinatal mood and anxiety disorder. Family J. 2023;31(2):237–44. 10.1177/10664807221147023. 10.1177/10664807221147023

[11] Sanger C, Iles JE, Andrew CS, Ramchandani PG. Associations between postnatal maternal depression and psychological outcomes in adolescent offspring: a systematic review. Archives Women’s Mental Health. 2014;18(2):147–62. 10.1007/s00737-014-0463-2. 10.1007/s00737-014-0463-2 25269760

[12] Ahun MN, Consoli A, Pingault J-B, Falissard B, Battaglia M, Boivin M, Tremblay RE, Côté SM. Maternal depression symptoms and internalising problems in the offspring: the role of maternal and family factors. Eur Child Adolesc Psychiatry. 2017;27(7):921–32. 10.1007/s00787-017-1096-6. 29273860 10.1007/s00787-017-1096-6

[13] Aoyagi S-S, Takei N, Nishimura T, Nomura Y, Tsuchiya KJ. Association of late-onset postpartum depression of mothers with expressive language development during infancy and early childhood: the HBC study. PeerJ. 2019;7:e6566. 10.7717/peerj.6566. 30863683 10.7717/peerj.6566 PMC6408909

[14] Miller ML, Williams BM, McCabe JE, Williamson JA, King S, Laplante DP, Hart KJ, O’Hara MW. Perinatal anxiety and depressive symptoms and perception of child behavior and temperament in early motherhood. J Dev Origins Health Disease. 2020;12(3):513–22. 10.1017/s2040174420000781. 10.1017/s2040174420000781 32907691

[15] Horowitz JA, Goodman J. A longitudinal study of maternal postpartum depression symptoms. Res Theory Nurs Pract. 2004;18(2):149–63. 10.1891/rtnp.18.2.149.61285. 15553344 10.1891/rtnp.18.2.149.61285

[16] Klier CM, Rosenblum KL, Zeller M, Steinhardt K, Bergemann N, Muzik M. A multirisk approach to predicting chronicity of postpartum depression symptoms. Depress Anxiety. 2008;25(8):718–24. 10.1002/da.20419. 18729148 10.1002/da.20419 PMC3150733

[17] Vliegen N, Casalin S, Luyten P. The course of postpartum depression. Harv Rev Psychiatry. 2014;22(1):1–22. 10.1097/hrp.0000000000000013. 24394219 10.1097/hrp.0000000000000013

[18] Batalova J. Chinese immigrants in the United States. Migrationpolicy.org, 12 Jan. 2023, www.migrationpolicy.org/article/chinese-immigrants-united-states

[19] Vigod S, Sultana A, Fung K, Hussain-Shamsy N, Dennis C-L. A population-based study of postpartum mental health service use by immigrant women in Ontario, Canada. Can J Psychiatry. 2016;61(11):705–13. 10.1177/0706743716645285. 27310236 10.1177/0706743716645285 PMC5066549

[20] Tulli M, Salami B, Begashaw L, Meherali S, Yohani S, Hegadoren K. Immigrant mothers’ perspectives of barriers and facilitators in accessing mental health care for their children. J Transcult Nurs. 2020;31(6):104365962090281. 10.1177/1043659620902812. 10.1177/1043659620902812 32013750

[21] Li Q, Xue W, Gong W, Quan X, Li Q, Xiao L, Xu D, Caine ED, Poleshuck EL. Experiences and perceptions of perinatal depression among new immigrant Chinese parents: a qualitative study. BMC Health Serv Res. 2021;21(1). 10.1186/s12913-021-06752-2. 10.1186/s12913-021-06752-2 PMC8311906 34311719

[22] Cacioppo JT, Hawkley LC. Perceived social isolation and cognition. Trends Cogn Sci. 2009;13(10):447–54. 10.1016/j.tics.2009.06.005. 19726219 10.1016/j.tics.2009.06.005 PMC2752489

[23] Pantell M, Rehkopf D, Jutte D, Syme SL, Balmes J, Adler N. Social isolation: a predictor of mortality comparable to traditional clinical risk factors. Am J Public Health. 2013;103(11):2056–62. 10.2105/AJPH.2013.301261. 24028260 10.2105/AJPH.2013.301261 PMC3871270

[24] Li Y, Dong F, Kim M. Mental health among Chinese immigrants in the United States during the COVID-19 pandemic. J Transcult Nurs. 2022;33(4):104365962210853. 10.1177/10436596221085300. 10.1177/10436596221085300 35466803

[25] Huang ZJ, Wong FY, Ronzio CR, Yu SM. Depressive symptomatology and mental health help-seeking patterns of U.S.- and Foreign-born mothers. Matern Child Health J. 2006;11(3):257–67. 10.1007/s10995-006-0168-x 10.1007/s10995-006-0168-x 17171544

[26] Ahmed A, Stewart DE, Teng L, Wahoush O, Gagnon AJ. Experiences of immigrant new mothers with symptoms of depression. Archives Women’s Mental Health. 2008;11(4):295–303. 10.1007/s00737-008-0025-6. 10.1007/s00737-008-0025-6 18677438

[27] Singh S, McBride K, Kak V. Role of Social Support in examining acculturative stress and psychological distress among Asian American immigrants and three sub-groups: results from NLAAS. J Immigr Minor Health. 2015;17(6):1597–606. 10.1007/s10903-015-0213-1. 25910620 10.1007/s10903-015-0213-1

[28] Derr AS. Mental Health Service Use among immigrants in the United States: a systematic review. Psychiatric Serv. 2016;67(3):265–74. 10.1176/appi.ps.201500004. 10.1176/appi.ps.201500004 PMC5122453 26695493

[29] Dennis C-L, Merry L, Stewart D, Gagnon AJ. Prevalence, continuation, and identification of postpartum depressive symptomatology among refugee, asylum-seeking, non-refugee immigrant, and Canadian-born women: results from a prospective cohort study. Archives Women’s Mental Health. 2016;19(6):959–67. 10.1007/s00737-016-0633-5. 10.1007/s00737-016-0633-5 27185244

[30] Langford CPH, Bowsher J, Maloney JP, Lillis PP. Social support: a conceptual analysis. J Adv Nurs. 1997;25(1):95–100. 10.1046/j.1365-2648.1997.1997025095.x. 9004016 10.1046/j.1365-2648.1997.1997025095.x

[31] Drageset J, Springer. https://www.ncbi.nlm.nih.gov/books/NBK585650/. 36315712

[32] Yang F, Jiang Y. Heterogeneous influences of Social Support on Physical and Mental Health: evidence from China. Int J Environ Res Public Health. 2020;17(18):6838. 10.3390/ijerph17186838. 32962140 10.3390/ijerph17186838 PMC7558190

[33] Bedaso A, Adams J, Peng W, Sibbritt D. The association between social support and antenatal depressive and anxiety symptoms among Australian women. BMC Pregnancy Childbirth. 2021;21(1). 10.1186/s12884-021-04188-4. 10.1186/s12884-021-04188-4 PMC8532351 34686140

[34] Reis D, Krautter K, Hart A, Friese M. Heterogeneity in Mental Health Change during the COVID-19 pandemic in Germany: the role of social factors. Stress Health. 2022. 10.1002/smi.3181. 35778965 10.1002/smi.3181 PMC9350024

[35] Shorey S, Chan SW-C, Chong YS, He H-G. Maternal parental self-efficacy in newborn care and social support needs in Singapore: a correlational study. J Clin Nurs. 2013;23(15–16):2272–83. 10.1111/jocn.12507. 24372630 10.1111/jocn.12507

[36] Chae SY, Chae MH, Kandula S, Winter RO. Promoting improved social support and quality of life with the CenteringPregnancy^®^ group model of prenatal care. Archives Women’s Mental Health. 2016;20(1):209–20. 10.1007/s00737-016-0698-1. 10.1007/s00737-016-0698-1 27988822

[37] Barbosa-Leiker C, Smith CL, Crespi EJ, Brooks O, Burduli E, Ranjo S, Carty CL, Hebert LE, Waters SF, Gartstein MA. Stressors, coping, and resources needed during the COVID-19 pandemic in a sample of perinatal women. BMC Pregnancy Childbirth. 2021;21(1). 10.1186/s12884-021-03665-0. 10.1186/s12884-021-03665-0 PMC7920400 33648450

[38] White LK, Kornfield SL, Himes MM, Forkpa M, Waller R, Njoroge WFM, Barzilay R, Chaiyachati BH, Burris HH, Duncan AF, Seidlitz J, Parish-Morris J, Elovitz MA, Gur RE. The impact of postpartum social support on postpartum mental health outcomes during the COVID-19 pandemic. Archives Women’s Mental Health. 2023;1–11. 10.1007/s00737-023-01330-3. 10.1007/s00737-023-01330-3 PMC10238239 37268777

[39] Chen H-H, Hwang F-M, Lin L-J, Han K-C, Lin C-L, Chien L-Y. Depression and Social Support trajectories during 1 year Postpartum among Marriage-based immigrant mothers in Taiwan. Arch Psychiatr Nurs. 2016;30(3):350–5. 10.1016/j.apnu.2015.12.008. 27256940 10.1016/j.apnu.2015.12.008

[40] Chen SH, Zhang E, Liu CH, Wang LK. Depressive symptoms in Chinese immigrant mothers: relations with perceptions of social status and interpersonal support. Cult Divers Ethnic Minor Psychol. 2020;27(1):72–81. 10.1037/cdp0000343. 10.1037/cdp0000343 32352807

[41] Liu J, Hung P, Alberg AJ, Hair NL, Whitaker KM, Simon J, Taylor SK. Mental health among pregnant women with COVID-19-related stressors and worries in the United States. Birth (Berkeley Calif). 2021;48(4):470–9. 10.1111/birt.12554. 34008216 10.1111/birt.12554 PMC8239832

[42] Cella D, Riley W, Stone A, Rothrock N, Reeve B, Yount S, Amtmann D, Bode R, Buysse D, Choi S, Cook K, DeVellis R, DeWalt D, Fries JF, Gershon R, Hahn EA, Lai J-S, Pilkonis P, Revicki D, Rose M. The patient-reported outcomes Measurement Information System (PROMIS) developed and tested its first wave of adult self-reported health outcome item banks: 2005–2008. J Clin Epidemiol. 2010;63(11):1179–94. 10.1016/j.jclinepi.2010.04.011. 20685078 10.1016/j.jclinepi.2010.04.011 PMC2965562

[43] Hahn EA, DeVellis RF, Bode RK, Garcia SF, Castel LD, Eisen SV, Bosworth HB, Heinemann AW, Rothrock N, Cella D. Measuring social health in the patient-reported outcomes measurement information system (PROMIS): item bank development and testing. Qual Life Res. 2010;19(7):1035–44. 10.1007/s11136-010-9654-0. 20419503 10.1007/s11136-010-9654-0 PMC3138729

[44] Schalet BD, Pilkonis PA, Yu L, Dodds N, Johnston KL, Yount S, Riley W, Cella D. Clinical validity of PROMIS Depression, anxiety, and anger across diverse clinical samples. J Clin Epidemiol. 2016;73(119–127):119–27. 10.1016/j.jclinepi.2015.08.036. 26931289 10.1016/j.jclinepi.2015.08.036 PMC4928679

[45] Rothrock NE, Amtmann D, Cook KF. Development and validation of an interpretive guide for PROMIS scores. J Patient-Reported Outcomes. 2020;4(1). 10.1186/s41687-020-0181-7. 10.1186/s41687-020-0181-7 PMC7048882 32112189

[46] Kroenke K, Yu Z, Wu J, Kean J, O. Monahan P. Operating characteristics of PROMIS four-item depression and anxiety scales in primary care patients with chronic pain. Pain Med (Malden Mass). 2014;15(11):1892–901. 10.1111/pme.12537. 10.1111/pme.12537 PMC6283354 25138978

[47] Katz PP, Robinson P, Trupin L, Rush S, Helmick CG, Murphy LB, Lanata C, Criswell LA, Dall’Era M. Psychometric Evaluation of the National Institutes of Health Patient-Reported Outcomes Measurement Information System in a multiracial, multiethnic systemic Lupus Erythematosus Cohort. Arthritis Care Res. 2019;71(12):1630–9. 10.1002/acr.23797. 10.1002/acr.23797 PMC6482092 30354017

[48] Cai T, Wu F, Huang Q, Yu C, Yang Y, Ni F, Yuan C. Validity and reliability of the Chinese version of the patient-reported outcomes Measurement Information System adult profile-57 (PROMIS-57). Health Qual Life Outcomes. 2022;20(1). 10.1186/s12955-022-01997-9. 10.1186/s12955-022-01997-9 PMC9202169 35706033

[49] Huang W, Wu Q, Zhang Y, Tian C, Huang H, Huang S, Zhou Y, He J, Wang H. Preliminary evaluation of the Chinese version of the patient-reported outcomes measurement information system 29-item profile in patients with aortic dissection. Health Qual Life Outcomes. 2022;20(1). 10.1186/s12955-022-02000-1. 10.1186/s12955-022-02000-1 PMC9195330 35701761

[50] Cai T, Huang Q, Wu F, Xia H, Yuan C. Psychometric validation of the Chinese version of the PROMIS Social relationships Short forms. Nurs Open. 2021;9(1):394–401. 10.1002/nop2.1077. 34569191 10.1002/nop2.1077 PMC8685787

[51] Alegría M, Álvarez K, DiMarzio K. Immigration and Mental Health. Curr Epidemiol Rep. 2017;4(2):145–55. 10.1007/s40471-017-0111-2. 29805955 10.1007/s40471-017-0111-2 PMC5966037

[52] Van Malderen C, Amouzou A, Barros AJD, Masquelier B, Van Oyen H, Speybroeck N. Socioeconomic factors contributing to under-five mortality in sub-saharan Africa: a decomposition analysis. BMC Public Health. 2019;19(1):760. 10.1186/s12889-019-7111-8. 31200681 10.1186/s12889-019-7111-8 PMC6570834

[53] Bas-Sarmiento P, Saucedo-Moreno MJ, Fernández-Gutiérrez M, Poza-Méndez M. Mental Health in immigrants Versus native Population: a systematic review of the literature. Arch Psychiatr Nurs. 2017;31(1):111–21. 10.1016/j.apnu.2016.07.014. 28104048 10.1016/j.apnu.2016.07.014

[54] Straiton M, Grant JF, Winefield HR, et al. Mental health in immigrant men and women in Australia: the North West Adelaide health study. BMC Public Health. 2014;14:1111. 10.1186/1471-2458-14-1111. 25349060 10.1186/1471-2458-14-1111 PMC4228163

[55] Gong F, Xu J, Fujishiro K, Takeuchi D T. A life course perspective on migration and mental health among Asian immigrants: the role of human agency. Soc Sci Med. 2011;73(11):1618–26. 10.1016/j.socscimed.2011.09.014. 10.1016/j.socscimed.2011.09.014 22019368

[56] Takeuchi DT, Zane N, Hong S, Chae DH, Gong F, Gee GC, Walton E, Sue S, Alegría M. Immigration-related factors and mental disorders among Asian americans. Am J Public Health. 2007;97(1):84–90. 10.2105/AJPH.2006.088401. 17138908 10.2105/AJPH.2006.088401 PMC1716230

[57] Wu Y, Zhang C, Liu H, Duan C, Li C, Fan J, Li H, Chen L, Xu H, Li X, Guo Y, Wang Y, Li X, Li J, Zhang T, You Y, Li H, Yang S, Tao X, Xu Y. Perinatal depressive and anxiety symptoms of pregnant women during the coronavirus disease 2019 outbreak in China. Am J Obstet Gynecol. 2020;223(2):e2401–9. 10.1016/j.ajog.2020.05.009. 10.1016/j.ajog.2020.05.009 PMC7211756 32437665

[58] Zheng Z, Zhang R, Liu T, Cheng P, Zhou Y, Lu W, Xu G, So K-F, Lin K. The psychological impact of the Coronavirus Disease 2019 pandemic on pregnant women in China. Front Psychiatry. 2021;12(628835). 10.3389/fpsyt.2021.628835. 10.3389/fpsyt.2021.628835 PMC8282994 34276429

[59] Kerker BD, Willheim E, Weis JR. The COVID-19 pandemic: Implications for Maternal Mental Health and early Childhood Development. Am J Health Promotion. 2023;37(2):265–9. 10.1177/08901171221140641b. 10.1177/08901171221140641b 36646659

[60] Chentsova Dutton YE, Choi I-J, Choi E. Perceived parental support and adolescents’ positive self-beliefs and levels of Distress Across Four Countries. Front Psychol. 2020;11(353). 10.3389/fpsyg.2020.00353. 10.3389/fpsyg.2020.00353 PMC7079630 32218754

[61] Senturk V, Abas M, Dewey M, Berksun O, Stewart R. Antenatal depressive symptoms as a predictor of deterioration in perceived social support across the perinatal period: a four-wave cohort study in Turkey. Psychol Med. 2016;47(4):766–75. 10.1017/s0033291716002865. 27873558 10.1017/s0033291716002865 PMC5426317

[62] Hijazi HH, Alyahya MS, Abdi A, Alolayyan RM, Sindiani MN, Raffee AM, Baniissa LA, W. A., Marzouqi A, A. M. The impact of Perceived Social Support during pregnancy on Postpartum infant-focused anxieties: a prospective cohort study of mothers in Northern Jordan. Int J Women’s Health. 2021;13:973–89. 10.2147/IJWH.S329487. 34707417 10.2147/IJWH.S329487 PMC8544270

[63] Falah-Hassani K, Shiri R, Vigod S, Dennis CL. Prevalence of postpartum depression among immigrant women: a systematic review and meta-analysis. J Psychiatr Res. 2015;70:67–82. 10.1016/j.jpsychires.2015.08.010. 26424425 10.1016/j.jpsychires.2015.08.010

[64] Sentell T, Shumway M, Snowden L. Access to mental health treatment by English language proficiency and race/ethnicity. J Gen Intern Med 22 Suppl. 2007;2(Suppl 2):289–93. 10.1007/s11606-007-0345-7. 10.1007/s11606-007-0345-7 PMC2150610 17957413

[65] Guruge S, Thomson MS, George U, Chaze F. Social support, social conflict, and immigrant women’s mental health in a Canadian context: a scoping review. J Psychiatr Ment Health Nurs. 2015;22(9):655–67. 10.1111/jpm.12216. 26031541 10.1111/jpm.12216

[66] Kim W, Kreps GL, Shin CN. The role of social support and social networks in health information-seeking behavior among Korean americans: a qualitative study. Int J Equity Health. 2015;14:40. 10.1186/s12939-015-0169-8. 25927546 10.1186/s12939-015-0169-8 PMC4419489

[67] Schweitzer R, Melville F, Steel Z, Lacherez P. Trauma, post-migration living difficulties, and social support as predictors of psychological adjustment in resettled Sudanese refugees. Aust N Z J Psychiatry. 2006;40(2):179–87. 10.1080/j.1440-1614.2006.01766.x. 16476137 10.1080/j.1440-1614.2006.01766.x

[68] Lundsberg LS, Schwarz EB, Vilardo N, Yonkers KA, Gariepy AM. Clinical validation of PROMIS Global short form in pregnancy. Appl Res Qual Life. 2018;13(1):89–103. 10.1007/s11482-017-9507-x

[69] Lau Y. Traditional Chinese pregnancy restrictions, Health-Related Quality of Life and perceived stress among pregnant women in Macao, China. Asian Nurs Res. 2012;6(1):27–34. 10.1016/j.anr.2012.02.005. 10.1016/j.anr.2012.02.005 25030688

[70] Center TH. (2020, October 17). Pregnancy Care in Traditional Chinese Medicine. TCM Healing Center. https://www.tcmhealingcenter.com/post/pregnancy-care-in-traditional-chinese-medicine

[71] Uchida Y, Kitayama S, Mesquita B, Reyes JAS, Morling B. Is Perceived Emotional Support Beneficial? Well-being and Health in Independent and interdependent cultures. Pers Soc Psychol Bull. 2008;34(6):741–54. 10.1177/0146167208315157. 18359927 10.1177/0146167208315157

[72] Qi W, Liu Y, Lv H, Ge J, Meng Y, Zhao N, Zhao F, Guo Q, Hu J. Effects of family relationship and social support on the mental health of Chinese postpartum women. BMC Pregnancy Childbirth. 2022;22(1). 10.1186/s12884-022-04392-w. 10.1186/s12884-022-04392-w PMC8787939 35078423

[73] Wong ST, Wu A, Gregorich S, Pérez-Stable EJ. What type of Social Support influences Self-reported physical and Mental Health among Older Women? J Aging Health. 2014;26(4):663–78. 10.1177/0898264314527478. 24733751 10.1177/0898264314527478 PMC4197109

