# Supplementary material for: The associations between social support and mental health among Chinese immigrant pregnant and parenting women
Source: BMC Pregnancy Childbirth. 2024 Sep 6;24:583. doi: 10.1186/s12884-024-06765-9 (PMC11380345; doi:10.1186/s12884-024-06765-9)
Supplement: Supplementary file 1 — Supplementary Material 1 [file 12884_2024_6765_MOESM1_ESM.docx]

**Supporting Table 1.**

| a. Association between Potential Control Variables and Anxiety and Depression | | | |  |  |  |  |  |  |  |  |  |
| --- | --- | --- | --- | --- | --- | --- | --- | --- | --- | --- | --- | --- |
|  |  | **Anxiety** | | | | |  | **Depression** | | | | |
| **Potential Control variables** |  | **n** | **mean** | **L95%CI** | **U95%CI** | **p-value** |  | **n** | **mean** | **L95%CI** | **U95%CI** | **p-value** |
| **Age** |  |  |  |  |  |  |  |  |  |  |  |  |
| 18-29 |  | 136 | 54.75 | 53.14 | 56.37 | ref |  | 136 | 52.93 | 51.47 | 54.39 | ref |
| 30 and above |  | 338 | 57.68 | 56.72 | 58.64 | **0.00*** |  | 339 | 55.71 | 54.75 | 56.68 | **0.00*** |
|  |  |  |  |  |  |  |  |  |  |  |  |  |
| **Marital Status** |  |  |  |  |  |  |  |  |  |  |  |  |
| Married/Living with Partner |  | 452 | 55.53 | 54.64 | 56.43 | ref |  | 452 | 53.72 | 52.86 | 54.59 | ref |
| Separated/Divorced/Widowed/Single |  | 48 | 59.37 | 56.79 | 61.95 | **0.01*** |  | 49 | 57.82 | 55.53 | 60.12 | **0.00*** |
|  |  |  |  |  |  |  |  |  |  |  |  |  |
| **Have difficulties in paying bills or buying something** | |  |  |  |  |  |  |  |  |  |  |  |
| No difficulty at all |  | 124 | 50.45 | 48.90 | 51.99 | ref |  | 124 | 48.35 | 46.87 | 49.83 | ref |
| Any level of difficulty |  | 334 | 58.26 | 57.27 | 59.25 | **0.00*** |  | 334 | 56.41 | 55.47 | 57.34 | **0.00*** |
|  |  |  |  |  |  |  |  |  |  |  |  |  |
| **In general, would you say your health is:** |  |  |  |  |  |  |  |  |  |  |  |  |
| Good and above |  | 432 | 54.53 | 53.67 | 55.40 | ref |  | 431 | 52.80 | 51.97 | 53.63 | ref |
| Poor/fair |  | 72 | 65.08 | 63.48 | 66.69 | **0.00*** |  | 73 | 62.97 | 61.41 | 64.54 | **0.00*** |
|  |  |  |  |  |  |  |  |  |  |  |  |  |
| **Education** |  |  |  |  |  |  |  |  |  |  |  |  |
| Less than high school degree |  | 203 | 55.61 | 54.13 | 57.10 | ref |  | 202 | 54.06 | 52.68 | 55.44 | ref |
| High school degree and above |  | 275 | 56.50 | 55.46 | 57.54 | 0.34 |  | 277 | 54.55 | 53.51 | 55.59 | 0.58 |
|  |  |  |  |  |  |  |  |  |  |  |  |  |
| **Speaks English at home** |  |  |  |  |  |  |  |  |  |  |  |  |
| No |  | 478 | 55.99 | 55.11 | 56.86 | ref |  | 479 | 54.21 | 53.38 | 55.04 | ref |
| Yes |  | 38 | 55.62 | 52.97 | 58.27 | 0.79 |  | 38 | 53.73 | 50.71 | 56.75 | 0.76 |
|  |  |  |  |  |  |  |  |  |  |  |  |  |
| **Years have been living in the U.S.  (Among those who not born in the U.S.)** |  |  |  |  |  |  |  |  |  |  |  |  |
| Less than 10 years |  | 243 | 57.42 | 56.29 | 58.54 | ref |  | 244 | 55.34 | 54.25 | 56.43 | ref |
| 10 years and above |  | 176 | 57.69 | 56.36 | 59.02 | 0.76 |  | 177 | 55.78 | 54.40 | 57.17 | 0.62 |
|  |  |  |  |  |  |  |  |  |  |  |  |  |
| b. Association between Potential Independent Variables and Anxiety and Depression | | | |  |  |  |  |  |  |  |  |  |
|  |  | **Anxiety** | | | | |  | **Depression** | | | | |
| **Potential Independent variables** |  | **n** | **r** | **L95%CI** | **U95%CI** | **p-value** |  | **n** | **r** | **L95%CI** | **U95%CI** | **p-value** |
|  |  |  |  |  |  |  |  |  |  |  |  |  |
| **Informational Support** |  | 500 | -0.05 | -0.14 | 0.04 | 0.28 |  | 500 | -0.08 | -0.17 | 0.01 | 0.08 |
|  |  |  |  |  |  |  |  |  |  |  |  |  |
| **Instrumental Support** |  | 499 | -0.19 | -0.27 | -0.11 | **0.00*** |  | 499 | -0.24 | -0.32 | -0.16 | **0.00*** |
|  |  |  |  |  |  |  |  |  |  |  |  |  |
| **Emotional Support** |  | 499 | -0.13 | -0.22 | -0.05 | **0.00*** |  | 499 | -0.18 | -0.27 | -0.10 | **0.00*** |

*Significant at the p ≤ 0.05 level.

| c. Association between Potential Control Variables and Support | | |  |  |  |  |  |  |  |  |  |  |  |  |  |  |  |
| --- | --- | --- | --- | --- | --- | --- | --- | --- | --- | --- | --- | --- | --- | --- | --- | --- | --- |
|  | **Instrumental Support** | | | | |  | **Emotional Support (PROMIS) - tscore** | | | | |  | **Informational Support** | | | | |
| **Potential Control variables** | **n** | **mean** | **L95%CI** | **U95%CI** | **p-value** |  | **n** | **mean** | **L95%CI** | **U95%CI** | **p-value** |  | **n** | **mean** | **L95%CI** | **U95%CI** | **p-value** |
| **Age** |  |  |  |  |  |  |  |  |  |  |  |  |  |  |  |  |  |
| 18-29 | 129 | 49.07 | 47.40 | 50.73 | ref |  | 131 | 45.33 | 44.05 | 46.62 | ref |  | 131 | 44.07 | 42.70 | 45.45 | ref |
| 30 and above | 336 | 46.71 | 45.73 | 47.69 | **0.02*** |  | 335 | 43.72 | 42.88 | 44.56 | **0.04*** |  | 335 | 43.12 | 42.22 | 44.03 | 0.26 |
|  |  |  |  |  |  |  |  |  |  |  |  |  |  |  |  |  |  |
| **Marital Status** |  |  |  |  |  |  |  |  |  |  |  |  |  |  |  |  |  |
| Married/Living with Partner | 445 | 47.68 | 46.85 | 48.52 | ref |  | 444 | 44.62 | 43.90 | 45.33 | ref |  | 446 | 43.79 | 43.02 | 44.56 | ref |
| Separated/Divorced/Widowed/Single | 46 | 41.66 | 38.70 | 44.63 | **0.00*** |  | 46 | 40.86 | 38.58 | 43.14 | **0.00*** |  | 46 | 39.65 | 37.14 | 42.15 | **0.00*** |
|  |  |  |  |  |  |  |  |  |  |  |  |  |  |  |  |  |  |
| **Have difficulties in paying bills or buying something** |  |  |  |  |  |  |  |  |  |  |  |  |  |  |  |  |  |
| No difficulty at all | 126 | 51.58 | 49.66 | 53.50 | ref |  | 124 | 46.55 | 45.01 | 48.10 | ref |  | 124 | 46.07 | 44.27 | 47.88 | ref |
| Any level of difficulty | 331 | 46.03 | 45.17 | 46.90 | **0.00*** |  | 330 | 43.53 | 42.77 | 44.28 | **0.00*** |  | 330 | 42.69 | 41.91 | 43.48 | **0.00*** |
|  |  |  |  |  |  |  |  |  |  |  |  |  |  |  |  |  |  |
| **In general, would you say your health is:** | |  |  |  |  |  |  |  |  |  |  |  |  |  |  |  |  |
| Good and above | 421 | 47.69 | 46.79 | 48.59 | ref |  | 421 | 44.88 | 44.12 | 45.64 | ref |  | 422 | 43.98 | 43.17 | 44.79 | ref |
| Poor/fair | 72 | 44.16 | 42.47 | 45.85 | **0.00*** |  | 71 | 40.95 | 39.84 | 42.07 | **0.00*** |  | 70 | 41.02 | 39.59 | 42.46 | **0.00*** |
|  |  |  |  |  |  |  |  |  |  |  |  |  |  |  |  |  |  |
| **Education** |  |  |  |  |  |  |  |  |  |  |  |  |  |  |  |  |  |
| Less than high school degree | 199 | 45.66 | 44.45 | 46.88 | ref |  | 196 | 43.32 | 42.31 | 44.32 | ref |  | 199 | 43.33 | 42.25 | 44.41 | ref |
| High school degree and above | 271 | 48.06 | 46.93 | 49.19 | **0.00*** |  | 273 | 44.89 | 43.92 | 45.86 | **0.03*** |  | 272 | 43.51 | 42.45 | 44.56 | 0.82 |
|  |  |  |  |  |  |  |  |  |  |  |  |  |  |  |  |  |  |
| **Speaks English at home** |  |  |  |  |  |  |  |  |  |  |  |  |  |  |  |  |  |
| No | 466 | 46.74 | 45.92 | 47.56 | ref |  | 467 | 43.78 | 43.09 | 44.47 | ref |  | 469 | 43.09 | 42.35 | 43.83 | ref |
| Yes | 38 | 51.83 | 48.39 | 55.28 | **0.01*** |  | 38 | 48.59 | 45.86 | 51.33 | **0.00*** |  | 38 | 46.63 | 43.51 | 49.75 | **0.03*** |
|  |  |  |  |  |  |  |  |  |  |  |  |  |  |  |  |  |  |
| **Years have been living in the U.S.  (Among those who not born in the U.S.)** |  |  |  |  |  |  |  |  |  |  |  |  |  |  |  |  |  |
| Less than 10 years | 239 | 47.86 | 46.78 | 48.93 | ref |  | 242 | 45.11 | 44.23 | 45.99 | ref |  | 241 | 44.17 | 43.22 | 45.12 | ref |
| 10 years and above | 174 | 47.48 | 46.02 | 48.94 | 0.68 |  | 173 | 43.97 | 42.80 | 45.13 | 0.12 |  | 172 | 43.16 | 41.93 | 44.39 | 0.20 |

*Significant at the p ≤ 0.05 level.

| **Supporting Table 2.** | | | | | | | | | | | | | |
| --- | --- | --- | --- | --- | --- | --- | --- | --- | --- | --- | --- | --- | --- |
| Multivariable Analysis: Examining Anxiety as an Outcome (Independent Variable – Informational Support) | | | | | | | | | | | | | |
| **Independent and Control Variables** |  | **Anxiety – Model 3 (N=403)** | | | | | | | | | | | |
|  |  | (Independent Variable: Informational Support) | | | | | | | | | | | |
|  |  | **Step 1 (Main Effect)** | | | | |  | **Step 2 (Interaction Effect)** | | | | | |
|  |  | **β** | **p-value** | **Adj. p-value** | **L95% CI** | **U95% CI** |  | **β** | **p-value** | **Adj. p-value** | **L95% CI** | **U95% CI** |  |
| **Age:** 30 or older ^a^ |  | 1.82 | **0.04ˆ** | 0.06 | 0.06 | 3.58 |  | 1.88 | **0.04ˆ** | 0.08 | 0.12 | 3.65 |  |
|  |  |  |  |  |  |  |  |  |  |  |  |  |  |
| **Marital status:** Separated/Divorced/Widowed/Single ^b^ |  | 3.79 | **0.01ˆ** | **0.02*** | 1.17 | 6.40 |  | 3.91 | **0.00ˆ** | **0.00*** | 1.29 | 6.53 |  |
|  |  |  |  |  |  |  |  |  |  |  |  |  |  |
| **Have difficulties in paying bills or buying something:** Any level of difficulty ^c^ |  | 5.99 | **0.00ˆ** | **0.00*** | 4.17 | 7.80 |  | 5.90 | **0.00ˆ** | **0.00*** | 4.08 | 7.72 |  |
|  |  |  |  |  |  |  |  |  |  |  |  |  |  |
| **General Health:** Poor and fair ^d^ |  | 8.05 | **0.00ˆ** | **0.00*** | 5.90 | 10.20 |  | 8.05 | **0.00ˆ** | **0.00*** | 5.90 | 10.20 |  |
|  |  |  |  |  |  |  |  |  |  |  |  |  |  |
| **Pregnancy status:** Pregnant ^e^ |  | 2.34 | **0.02ˆ** | **0.04*** | 0.43 | 4.24 |  | 2.36 | **0.02ˆ** | 0.06 | 0.46 | 4.27 |  |
|  |  |  |  |  |  |  |  |  |  |  |  |  |  |
| **Informational Support** |  | 0.02 | 0.67 | 0.67 | -0.08 | 0.12 |  | 0.04 | 0.43 | 0.52 | -0.06 | 0.15 |  |
|  |  |  |  |  |  |  |  |  |  |  |  |  |  |
| **Interaction: Support X Pregnancy Status:** Pregnant ^f^ |  |  |  |  |  |  |  | -0.14 | 0.30 | 0.42 | -0.40 | 0.12 |  |
|  |  |  |  |  |  |  |  |  |  |  |  |  |  |

^a^Reference group: Ages18-29

^b^Reference group: Married or living with partner

^c^Reference group: No difficulty at all

^d^Reference group: Good and above

^e,f^Reference group: Non-pregnant

ˆ Significant at p ≤ 0.05 level based on original p-value

* Significant at p ≤ 0.05 level based on adjusted p-value (Benjamini-Hochberg procedure)

| **Supporting Table 3.** | | | | | | | | | | | | | |
| --- | --- | --- | --- | --- | --- | --- | --- | --- | --- | --- | --- | --- | --- |
| Multivariable Analysis: Examining Depression as an Outcome (Independent Variable – Informational Support) | | | | | | | | | | | | | |
|  |  | **Depression – Model 3 (N=402)** | | | | | | | | | | | |
|  |  | (Independent Variable: Informational Support) | | | | | | | | | | | |
|  |  | **Step 1 (Main Effect)** | | | | |  | **Step 2 (Interaction Effect)** | | | | | |
| **Independent and Control Variables** |  | **β** | **p-value** | **Adj. p-value** | **L95% CI** | **U95% CI** |  | **β** | **p-value** | **Adj. p-value** | **L95% CI** | **U95% CI** |  |
| **Age:** 30 or older ^a^ |  | 1.44 | 0.10 | 0.18 | -0.28 | 3.17 |  | 1.42 | 0.11 | 0.22 | -0.31 | 3.14 |  |
|  |  |  |  |  |  |  |  |  |  |  |  |  |  |
| **Marital status:** Separated/Divorced/Widowed/Single ^b^ |  | 4.11 | **0.00ˆ** | **0.00*** | 1.55 | 6.66 |  | 4.05 | **0.00ˆ** | **0.00*** | 1.49 | 6.62 |  |
|  |  |  |  |  |  |  |  |  |  |  |  |  |  |
| **Have difficulties in paying bills or buying something:** Any level of difficulty ^c^ |  | 6.21 | **0.00ˆ** | **0.00*** | 4.44 | 7.99 |  | 6.25 | **0.00ˆ** | **0.00*** | 4.47 | 8.04 |  |
|  |  |  |  |  |  |  |  |  |  |  |  |  |  |
| **General Health:** Poor and fair ^d^ |  | 7.65 | **0.00ˆ** | **0.00*** | 5.55 | 9.74 |  | 7.65 | **0.00ˆ** | **0.00*** | 5.55 | 9.75 |  |
|  |  |  |  |  |  |  |  |  |  |  |  |  |  |
| **Pregnancy status:** Pregnant ^e^ |  | 0.59 | 0.53 | 0.64 | -1.27 | 2.46 |  | 0.58 | 0.54 | 0.65 | -1.28 | 2.45 |  |
|  |  |  |  |  |  |  |  |  |  |  |  |  |  |
| **Informational Support** |  | -0.02 | 0.63 | 0.63 | -0.12 | 0.07 |  | -0.03 | 0.53 | 0.65 | -0.14 | 0.07 |  |
|  |  |  |  |  |  |  |  |  |  |  |  |  |  |
| **Interaction: Support X Pregnancy Status:** Pregnant ^f^ |  |  |  |  |  |  |  | 0.06 | 0.64 | 0.64 | -0.20 | 0.32 |  |
|  |  |  |  |  |  |  |  |  |  |  |  |  |  |

^a^Reference group: Ages18-29

^b^Reference group: Married or living with partner

^c^Reference group: No difficulty at all

^d^Reference group: Good and above

^e,f^Reference group: Non-pregnant

ˆ Significant at p ≤ 0.05 level based on original p-value

* Significant at p ≤ 0.05 level based on adjusted p-value (Benjamini-Hochberg procedure)
